# Pharmacological treatment strategies to manage precipitated withdrawal following the administration of buprenorphine in opioid use disorder: A systematic review

**DOI:** 10.1111/add.70334

**Published:** 2026-01-22

**Authors:** Emmert Roberts, Nicola Kalk, John Strang

**Affiliations:** ^1^ National Addiction Centre, Institute of Psychiatry, Psychology and Neuroscience (IoPPN), King's College London, and South London and the Maudsley NHS Foundation Trust London UK

**Keywords:** BPOW, buprenorphine, opioid use disorder, pharmacological management, precipitated withdrawal, systematic review

## Abstract

**Background and aims:**

There has been limited evidence synthesis examining the treatment of buprenorphine precipitated opioid withdrawal (BPOW). We aimed to conduct the first systematic review to assess the clinical utility of any pharmacological intervention in the management of BPOW.

**Methods:**

Systematic review searching Medline, Embase, PsychINFO and CENTRAL from the date of database inception to 26 August 2025 for studies of any design reporting any pharmacological intervention in the management of BPOW compared with any or no other interventions, using adult participants aged 18 or over receiving buprenorphine and experiencing BPOW. We planned to combine outcomes using random‐effects meta‐analysis; where this was not possible results were reported narratively. We considered two outcomes and extracted (1) any reported measure or description of the change in opioid withdrawal symptoms (OWS) and (2) the number of individuals retained in buprenorphine treatment. Outcome quality was assessed using the Grading of Recommendations Assessment, Development and Evaluation (GRADE) framework.

**Results:**

Forty‐three studies met inclusion criteria reporting on 137 participants. These comprised one pilot randomised controlled trial and 42 uncontrolled observational case series or case reports. Meta‐analysis was not possible, and all evidence was of *low* or *very low* quality. The currently available randomised evidence suggests that use of intravenous magnesium sulphate, in addition to symptomatic treatment with clonidine, paracetamol and diazepam, may statistically significantly reduce OWS when compared with symptomatic treatment alone. The currently available observational evidence suggests that treatment strategies which include additional doses of transmucosal buprenorphine may demonstrate higher rates of symptom control and treatment retention than strategies which do not include additional doses of transmucosal buprenorphine.

**Conclusions:**

There is a paucity of research into pharmacological management of buprenorphine precipitated opioid withdrawal. The limited *very low* to *low* quality evidence suggests treatment regimens that include magnesium sulphate and additional doses of transmucosal buprenorphine are potentially the most salient current options and avenues for future research in the management of buprenorphine precipitated opioid withdrawal.

## INTRODUCTION

There are several scenarios in which transition from full opioid agonists to the partial agonist buprenorphine may be clinically desirable [1]. These include commencement of buprenorphine as an opioid agonist therapy (OAT) in people with opioid use disorder (OUD) and switching to buprenorphine from other forms of OAT (e.g. methadone) because of a favourable safety profile [1]. However, administration of buprenorphine in the presence of a full agonist can lead to the development of buprenorphine precipitated opioid withdrawal (BPOW). This is operationally defined as an increase in opioid withdrawal symptoms (OWS) following buprenorphine administration and can manifest as severe distress because of the rapid displacement of full agonists from μ‐opioid receptors following exposure to a higher affinity partial agonist [2].

Despite low overall incidence (range = 0%–13%) [3], fear of developing BPOW among people with OUD and prescribers remains a significant barrier to commencing or switching to buprenorphine treatment, particularly among those currently receiving methadone [4, 5]. Poorly managed BPOW may also reduce the likelihood of successful initiation and retention in buprenorphine treatment [6]. Although there have been recent advances to mitigate the risk of developing BPOW, including direct induction onto long‐acting injectable buprenorphine [7] and ‘microdose’ induction regimens compared to the conventional practice of allowing time for the development of sufficient OWS following full agonist cessation, there has been limited evidence synthesis addressing the management of BPOW itself [6]. To our knowledge, no previous systematic review exists to evaluate any individual pharmacological agent or treatment strategy in the management of BPOW. Although multiple guidelines endorse treatment with additional doses of buprenorphine or symptomatic medication regimens, management strategies are highly variable, typically based on clinical consensus and often either do not reference empirical data sources or cite only single case reports [8, 9, 10].

Internationally, there are also concerns that higher rates of BPOW may be observed, despite standard periods of abstinence, because of the increased use of highly lipophilic fentanyl analogues [3, 11]. Suboptimal BPOW management may impact longer‐term individual‐ and population‐level treatment outcomes via reducing an individual's willingness to subsequently consider buprenorphine as a treatment option and fostering negative perceptions of buprenorphine among people who use drugs [12]. The demographic and clinical profile of people with OUD is changing with higher numbers of people dependent on synthetic opioids, more individuals of advancing age using methadone and expansions in the availability of long‐acting injectable buprenorphine [13]. These may lead to an increasing demand for buprenorphine and more opportunistic and complex initiations in a variety of settings [14]. As such, a more robust understanding of the available evidence for pharmacological treatment of BPOW is currently vital and necessary to improve knowledge among prescribers and minimise distressing OWS. Optimised BPOW management may also improve treatment retention, reducing the mortality risk associated with OAT discontinuation [15].

To address this gap, we aimed to conduct a systematic review to assess the clinical utility of any pharmacological treatment strategy in the management of BPOW. Additionally, we planned to use Grading of Recommendations Assessment, Development, and Evaluation (GRADE) methodology to assess the quality of the evidence and the strength of any resultant recommendations.

## METHODS

This study is reported according to the Preferred Reporting Items for Systematic Reviews and Meta‐Analyses (PRISMA) guidelines [16] the checklist available as Table S6 in the online supplementary material, with the review protocol pre‐registered on PROSPERO (registration number: CRD420251137026).

### Search strategy

We searched Medline, Embase, PsychINFO and the Cochrane Central Register of Controlled Trials (CENTRAL) from the date of database inception to 26 August 2025. The complete search strategy can be found as Figure S1 the online Supporting information.

### Study selection

Two authors initially assessed titles and abstracts and reviewed the full text of remaining articles for inclusion. Any discrepancy was resolved by discussion and where agreement could not be reached a third author was consulted. All relevant references were checked for additional citations.

The review protocol can be found as Table S1. We included studies of any design where any pharmacological intervention had been specifically used to manage symptoms of BPOW in adults. Full descriptions of each included study's population, intervention/s and, where applicable, comparison/s can be found in Tables 1 and 2.

**TABLE 1:** Characteristics of included randomised controlled trials.

Study ID	Population							Intervention	Comparison	Outcomes					
	Setting	Definition of precipitated withdrawal	Baseline opioid use	Administered BPN dose (mg) mean (SD)	F:M	Age (years) mean (SD)	Country	Description no. randomised (*n*)	Description no. randomised (*n*)	COWS score, mean (SD)					
										30 min post‐treatment			2 h post‐treatment		
										I	C	*P* value	I	C	*P* value
Moshiri *et al*. [17]	ED	'Patients who had symptoms of precipitated opioid withdrawal’	Opium (30%) Heroin (15%) Methadone (55%)	2.2 (1.4)	1:39	34.5 (7.4)	Iran	Magnesium sulphate (MgSO_4_) 3 g over 20 min then 10 mg/kg/h up to 2^O^ conventional therapies *n* = 20	Conventional therapies: clonidine 0.1 mg PO 1° PRN; diazepam 10 mg IV infusions; paracetamol 1 g IV *n* = 20	11.2 (2.9)	14.4 (3.3)	0.002	3.2 (1.6)	11.3 (3.3)	<0.001

Notes: COWS is an 11 item clinician‐rated scale with scores 0–47; higher scores indicate more severe withdrawal symptoms [18].

Abbreviations: BPN, buprenorphine; C, control; COWS, Clinical Opiate Withdrawal Scale; ED, emergency department; F, female; I, intervention; IV, intravenous; M, male; mg, milligram; PO, Per os (oral); PRN, pro re nata (as required).

**TABLE 2:** Characteristics of included observational case descriptions, case series and case reports.

Study ID	Study design	Population								Pharmacological interventions	Outcomes	
											Change in opioid withdrawal symptoms	Retained in BPN treatment? (*n*)
		Setting	Definition/reported symptoms of precipitated withdrawal	Baseline opioid use	Administered BPN formulation/dose	*n*	F:M	Age (years)	Country			
Hermenau *et al*. [19]	Case report	Hospital in‐patient	‘Severe distress, hallucinations, restlessness and confusion’	Methadone 210 mg PO od	BPN 8 mg s/l	1	0:1	37	USA	Hydromorphone 1 mg IV Haloperidol 5 mg IM Lorazepam 3 mg IV Oxycodone 5 mg PO 6°	‘Within 24 hours, the patient's withdrawal symptoms were controlled’	Not retained
Gbujie *et al*. [20]	Case report	Hospital in‐patient	↑COWS 1 → 15 + ↑COWS 2 → 12	Fentanyl 1.5 g IN weekly	BPN/NX 16 mg s/l +BPN/NX 24 mg s/l	1	0:1	72	USA	Hydromorphone 2 mg IV +Hydromorphone 2 mg IV ×2 BPN 8 mg s/l Lorazepam 2 mg IV Clonidine PO 0.1 mg Ketamine IV 0.3 mg/kg Haloperidol 5 mg IV ×2	Initially ↓COWS → 5 → ↑COWS → 20 → ‘Acute delirium and hallucinations following ketamine’ → ‘By day 6 mental status had returned to baseline’	Retained
Checkley *et al*. [21]	Case descriptions: 2 cases within retrospective cohort study	ED	(1) ‘Worsening of…withdrawal symptoms’ (2) ↑COWS 5 → 26	‘Patients who used fentanyl’	(1) BPN 1 mg s/l (2) BPN 8 mg s/l	2	2:0	NR	USA	(1) Hydromorphone infusion (2) BPN patch Clonidine Dexmedetomidine Fentanyl infusion Ondansetron	NR	NR
Chakraborty *et al*. [22]	Case series: 10 cases	ED	‘Severe opioid withdrawal, all suspected to be precipitated’	Multiple	Doses of BPN and BPN/NX ranging from ‘unknown’ dose to 16 mg s/l	10	3:7	Median 35 (IQR = 26)	USA	Hydromorphone IV (all) Benzodiazepines (n = 3) Antipsychotics (n = 2) Clonidine (n = 2) BPN (n = 3) Antiemetic (n = 1)	All improved opioid withdrawal symptoms following hydromorphone	9/10 retained
O'Conor *et al*. [23]	Case descriptions: 2 cases within a case series	Hospital in‐patient	NR	NR	(1) BPN 8 mg s/l (2) BPN 16 mg s/l	2	NR	NR	USA	Methadone	NR	Both patients started on LAIB
Koenigs*et al*. [24]	Case description: 1 case within a case series	Hospital in‐patient	NR	‘Illicit fentanyls, methadone stabilisation’	Microinduction regimen commencing with BPN buccal film 225 μg bd	1	1:0	39	USA	Hydroxyzine Clonazepam Clonidine Quetiapine	NR	Retained
Yakey *et al*. [25]	Case series: 2 cases	ED	NR	(1) ‘Daily fentanyl use’ (2) Methadone 95 mg od	(1) BPN 8 mg s/l (2) BPN 8 mg s/l	2	0:2	(1) 52 (2) 36	USA	(1) BPN 32 mg s/l Lorazepam 3 mg PO Ondansetron 4 mg (2) BPN 40 mg s/l Diazepam 5 mg IV Diazepam 5 mg PO +BPN 16 mg s/l ×2	(1) ‘Resolution of symptoms’ (2) ‘Rapid improvement’	(1) NR (2) Retained
Vercollone *et al*. [26]	Case report	Hospital in‐patient	↑COWS ≥6	Fentanyl IV 2 g/day	BPN/NX 2 mg s/l	1	0:1	26	USA	BPN 24 mg s/l +BPN 8 mg s/l ×2 Chlordiazepoxide 375 mg PO lorazepam 8 mg IV Hydromorphone 4 mg IV	Persistent COWS → 25 Self‐discharge	Not retained
Terasaki and Brady [27]	Case report	Hospital in‐patient	‘Based on his mental status’	‘Smoking fentanyl’	BPN 8 mg s/l	1	0:1	30	USA	BPN 16 mg s/l Diazepam 20 mg PO od Haloperidol 10 mg IM Clonidine + methadone 20 mg PO	‘Symptoms improved’ following methadone	Not retained
Spadaro *et al*. [28]	Case descriptions: 9 cases within a case series	Hospital in‐patient	↑COWS ≥6 within 2 hours of BPN and/or ‘documentation specifically describing acutely worsening withdrawal symptoms’	Fentanyl/heroin/oxycodone	BPN 2‐8 mg s/l	9	NR	NR	USA	BPN (*n* = 7) Loperamide (*n* = 3) Ondansetron (*n* = 2) Paracetamol/NSAIDs (*n* = 2) Oxycodone (*n* = 1) Clonidine (*n* = 4) Benzodiazepines (*n* = 7) Hydroxyzine (*n* = 2) Hydromorphone (*n* = 1) Dicyclomine (*n* = 3)	Five patients' symptoms improved between 4 up to 28 hours; 2 did not improve; 1 died; 1 no results documented	4/9 retained
Dash *et al*. [29]	Case series: 2 cases	NR	(1) ↑COWS → 27 (2) ↑COWS → 16	Fentanyl	(1) BPN/NX 4 mg film (2) BPN/NX 2 mg s/l	2	1:1	(1) 58 (2) 40	USA	(1) BPN 8 mg s/l Clonidine 0.2 mg +BPN 2 mg s/l ×2 Clonidine 0.2 mg Diazepam 10 mg PO +Droperidol 1.25 mg IV BPN 8 mg s/l Clonidine 0.3 mg (2) Diazepam 5 mg PO Ondansetron Droperidol 2.5 mg IV	(1) ‘Symptoms markedly started improving was after droperidol administration’ (2) ‘Symptoms improved rapidly after administration of…droperidol’	(1) Retained (2) NR
Church *et al*. [30]	Case report	ED	↑COWS → 6	Heroin 0.75–1.5 g/day in + an unknown dose of illicit methadone	An unknown dose of illicit BPN/NX	1	0:1	55	USA	Methadone 20 mg PO	*‘*Improvement in his withdrawal symptoms’	Retained on methadone
Christian *et al*. [31]	Case report	ED	↑COWS → 36	Smoking 5 bags of fentanyl per day	BPN/NX 16 mg s/l	1	0:1	32	USA	Clonidine 0.2 mg Diazepam 10 mg Haloperidol 2 mg IV Haloperidol 5 mg PO +Ketamine infusion 0.3 mg/kg ×2 BPN 16 mg s/l ×2	Symptoms improved; ↓COWS → 18; Withdrawal symptoms completely resolved within 8 hours of second infusion	Retained
Shearer *et al*. [32]	Case series: 3 cases	Outpatient clinic	(1) ‘Increased severity of diaphoresis, lacrimation, rhinorrhoea and diarrhoea’ (2) ‘Sudden worsening of withdrawal symptoms, including akathisia, myalgias and diaphoresis’ (3) ‘Increased restlessness and anxiety’	(1) Smoking 1 g fentanyl/day (2) Slow‐release oral morphine 900 mg/day and fentanyl 0.1 g/day (3) Smoking 0.25 g fentanyl/day	(1) BPN/NX 2 mg s/l (2) BPN/NX 2 mg s/l + 2 mg s/l (3) BPN/NX 2 mg s/l	3	0:3	(1) 49 (2) 41 (3) 42	Canada	(1) Hydromorphone Clonidine (2) BPN 6 mg s/l (3) BPN 16 mg s/l Gabapentin 600 mg PO Quetiapine 50 mg PO +BPN 8 mg s/l +BPN 6 mg s/l	(1) ‘Symptoms were challenging to bring under control’ (2) ‘Symptoms of opioid withdrawal had improved significantly after completing the full course of BUP/NX’ (3) ‘Significant improvement in his symptoms’	(1) Not retained (2) Not retained (3) Retained
Quattlebaum *et al*. [33]	Case report	Home	‘Worsening of her withdrawal symptoms including anxiety, sweatiness, restless legs, chills and pain’	Oxycodone ER 20 mg PO tds Oxycodone 10 mg PO 6° PRN	BPN/NX 4 mg s/l +BPN/NX 4 mg s/l	1	1:0	‘Middle aged’	USA	BPN 8 mg s/l Paracetamol 1 g PO Antiemetics Clonidine +BPN 4 mg s/l	↓COWS 25 → 13 → 0	Retained
Heeney *et al*. [34]	Case series: 10 cases	Community (*n* = 3) ED (*n* = 7)	‘Precipitated withdrawal’	‘Daily fentanyl (90%) or heroin (60%) use’	‘Buprenorphine’	10	NR	Mean 34.3	USA	‘High dose’ BPN +Ketamine infusion 0.3–1 mg/kg	‘Significant improvement in their symptoms as measured by COWS’	‘8/10 remained engaged in treatment’
Comstock *et al*. [35]	Case series: 4 cases	Home → ED	‘Recalcitrant hyperactive delirium’	‘Up to 8 ‘M30 blues’ fentanyl daily’	BPN/NX 16 mg s/l	4	0:1	59	USA	‘High dose’ hydromorphone infusion	‘Symptom control’	NR
		ED	‘Worsening myalgia and restlessness’	‘Smoking up to 10 ‘blues’ fentanyl daily’	BPN/NX 8 mg s/l +BPN/NX 24 mg s/l		0:1	49		Hydromorphone infusion	‘Difficult to manage until day 3 when a hydromorphone infusion was initiated’	
		Prison	‘Hyperactive delirium’	‘Smoking 20–30 ‘fentanyl tablets’ daily’	‘Buprenorphine’		1:0	39		‘Deep sedation’	‘Symptom control’	
		NR	‘Escalating agitation and delirium’	‘Insufflating’ 14–35 fentanyl ‘blues’ daily’	‘Buprenorphine’		1:0	27		Hydromorphone infusion	‘Course improved’	
Bormann *et al*. [36]	Case report	Outpatient clinic → ED	↑COWS → 27	‘Daily fentanyl use’	BPN/NX 8 mg s/l	1	0:1	21	USA	BPN 8 mg s/l +BPN 8 mg s/l +BPN 4 mg s/l ×3 +BPN 16 mg s/l ×3 Gabapentin 600 mg PO tds Lorazepam 2 mg PO	‘Improved with repeat dosing of buprenorphine’ ↓COWS 19 → 9	Retained
Berland *et al*. [37]	Case report	ED	‘Worsening withdrawal symptoms noted’	Loperamide 800 mg/day	BPN 32 mg s/l	1	0:1	42	USA	BPN 32 mg s/l BPN 1.8 mg IV Clonidine Diazepam	‘Decreased withdrawal symptoms’	Retained
Sherer *et al*. [38]	Case report	NR	‘Precipitated opioid withdrawal’	‘…opioid use disorder who insufflated six bags of heroin’	‘Crushed tablet of Subutex’	1	0:1	49	USA	BPN 24 mg s/l +BPN 0.3 mg IM	‘Attenuated the withdrawal symptoms’	NR
Rigney and Wiegand [39]	Case report	ED	‘Diffuse pain, cramping, muscle spasms, and myoclonic jerks’	Methadone 70 mg PO od oxymorphone 200 mg PO od	BPN 8 mg s/l +BPN 4 mg s/l	1	0:1	55	USA	BPN 4 mg s/l Midazolam Clonidine 0.2 mg Lorazepam 2 mg	‘Symptoms stabilized with repeated doses of clonidine/lorazepam’	Retained
Oakley *et al*. [40]	Case report	Outpatient clinic → ED	↑COWS 14 → 33	Heroin 0.1 g IV bd	BPN/NX 8 mg s/l	1	0:1	53	Australia	BPN 8 mg s/l Ondansetron 4 mg IV Diazepam 5 mg PO Buscopan 20 mg IV +BPN 8 mg s/l Paracetamol 1 g PO Metoclopramide 10 mg IM	↓COWS 22 → 13 → 3	Retained
Montalvo *et al*. [41]	Case report	Hospital in‐patient	↑COWS 4 → 16	Heroin and fentanyl use IV	BPN/NX 2 mg s/l	1	0:1	61	USA	Oxazepam 30 mg PO Clonidine Ondansetron Dicyclomine	‘Worsening nausea and vomiting with COWS between 8 and 11’	Retained
Jamshidi *et al*. [42]	Case report	Hospital in‐patient	‘Shortness of breath (SOB) and ongoing vomiting’	Methadone 40 mg PO od	BPN 4 mg s/l +BPN 4 mg s/l	1	1:0	65	Australia	BPN Diazepam	‘Her mentation improved’	NR
Herring *et al*. [43]	Case descriptions: 5 cases within a case series	ED	‘Clinician diagnosis of precipitated withdrawal or documentation of increasing severity of COWS within 1 hour’	NR	BPN 8 mg s/l (*n* = 4) BPN > 28 mg s/l (*n* = 1)	5	NR	NR	USA	BPN total 28 mg s/l	‘In all cases the patients were discharged in stable or improved conditions’	NR
Antoine *et al*. [44]	Case descriptions: 2 cases within a case series	NR	↑COWS 9 → 19 ↑COWS 9 → 35	‘4–5 capsules fentanyl intranasal per day’ ‘2–3 capsules of heroin/fentanyl per day IV’	BPN 4 mg s/l BPN 4 mg s/l	2	0:1 1:0	Between 41 and 55 Between 26 and 40	USA	BPN 4 mg s/l BPN 4 mg s/l	COWS ‘continued to increase’ ‘Continuation of severe withdrawal symptoms’	NR
												NR
LeSaint *et al*. [45]	Case description: 1 case within a case series	ED	‘Within an hour he developed restlessness, body aches, runny nose, gastrointestinal upset, anxiety, and gooseflesh skin’	‘Daily heroin insufflation’	BPN 8 mg s/l	1	0:1	54	USA	Ondansetron Ketorolac Lorazepam	‘Repeat COWS score was 6’	Not retained
Kinasz *et al*. [46]	Case report	Hospital in‐patient	↑COWS →11 → 27 ‘Increasingly agitated…his behaviour escalated to yelling and throwing furniture’	Methadone 90 mg PO od	BPN transdermal patch 20 μg per hour	1	0:1	‘Late 50s’	USA	Clonazepam 2 mg PO Clonidine Trazodone Ibuprofen +Haloperidol 5 mg PO Diphenhydramine 50 mg PO clonazepam 0.5 mg PO BPN patch 20 μg/hour +BPN 4 mg s/l Lorazepam 2 mg IM	‘Leg movements significantly decreased ‘↓COWS→5	Retained
Surmaitis *et al*. [47]	Case report	Drug rehab facility	‘Tachycardic, tachypneic and diaphoretic’	2–3 bags heroin/day	BPN 4 mg s/l	1	1:0	34	USA	BPN 4 mg s/l	‘No improvement of her symptoms’	NR
Lintzeris *et al*. [48]	Case descriptions: 3 cases within a prospective cohort study	Outpatient	↑ COWS of 6 or more points within 6 hours of the first BPPN/NX dose	Methadone 155 mg PO od	BPN/NX 8 mg s/l	3	NR	NR	Australia	BPN 32 mg s/l Paracetamol 1 g PO Metoclopramide 10 mg IM Methadone 40 mg PO bd	↓COWS 27 → 4	Retained in methadone treatment
		Hospital in‐patient		Methadone 75 mg PO od	BPN/NX 2 mg s/l					BPN 30 mg s/l Temazepam 20 mg PO Methadone 75 mg PO	↓COWS 17 → 7	
		Hospital in‐patient		Methadone 95 mg PO od	BPN/NX 2 mg s/l					BPN 14 mg s/l Hyoscine butylromide 20 mg PO Metoclopramide 10 mg IM Temazepam 20 mg +BPN 32 mg s/l	↓COWS 20 → 8	
Cortina *et al*. [49]	Case report	Hospital in‐patient	Post‐dose COWS score of >2 points than a preceding pre‐dose score	Methadone 180 mg PO od	BPN transdermal patch 20 μg per hour +BPN 1 mg s/l +BPN 1 mg s/l	1	0:1	34	Canada	Clonidine Olanzapine Lorazepam Dimenhydrinate	‘Patient's withdrawal symptoms improved’ ↓COWS 16 → 4	Retained
Oretti *et al*. [50]	Case description: 1 case within a case series	Hospital in‐patient	‘Became anxious and indicated that he was experiencing severe withdrawal’	Methadone 50 mg PO od + heroin	BPN/NX 4 mg s/l	1	0:1	39	United Kingdom	Methadone 25 mg PO	‘Did not wish to continue with transfer’	NR
Gangahar *et al*. [51]	Case report	Home → ED	‘Diaphoresis, chills and nausea’	Oxycodone 60 mg PO oxymorphone 10 mg PO + heroin	Illicit BPN/NX 2 mg s/l	1	0:1	21	USA	Lorazepam 1 mg ×2 Haloperidol 5 mg IV	‘Became drowsy but remained agitated, jerking, [and] confused’	Retained
Eggleston *et al*. [52]	Case report	ED	‘Agitated and combative with hallucinations’	Loperamide 400 mg PO od	BPN 12 mg s/l	1	0:1	30	USA	Lorazepam Isoproterenol infusion Propofol	‘Sedated’	NR
Wiegand [53]	Case report	Home → ED	‘Recurrent vomiting, tachycardic and hypertensive’	‘Methadone‐dependent’	‘Several’ illicit BPN/NX 8 mg tablets	1	0:1	61	USA	Fentanyl 200 μg IV increments 20 mg lorazepam	‘Did not improve his clinical status’	NR
Jutras‐Aswad *et al*. [54]	Case report	Hospital in‐patient	‘Anxiety, myalgia, flushing, lacrimation, chills, nausea and vomiting, diarrhoea’	Methadone 165 mg PO od	BPN 4 mg s/l	1	0:1	62	USA	+BPN 8 mg s/l per day	‘On the fourth day the patient was, comfortable and relieved of withdrawal symptoms’	Retained
Whitley *et al*. [55]	Case descriptions: 10 cases within a case series	Community	‘Acute worsening of opioid withdrawal symptoms immediately following initial dose’	Methadone (*n* = 9)	‘Most patients received’ BPN/NX 2 mg s/l	10	NR	NR	USA	‘Increased doses’ BPN Clonidine Loperamide Ibuprofen	NR	*‘6/10 discontinued treatment’*
Lee *et al*. [56]	Case descriptions: 5 cases within a prospective cohort study	Outpatient clinic	‘Any new or worsening opioid withdrawal symptoms not present or worsening with the initial doses’	Opioid dependence	BPN/NX 4 mg s/l	5	NR	NR	USA	‘Further buprenorphine doses’	‘Symptoms were described as mild or moderate, relieved by further buprenorphine doses, and resolved by induction day 3”	5/5 Retained
Ang‐Lee *et al*. [57]	Case description: 1 case within a case series	Hospital in‐patient	↑COWS 14 → 28	Heroin use	BPN/NX 24 mg s/l	1	NR	NR	USA	Chlordiazepoxide 75 mg Methocarbamol 1500 mg +Chlordiazepoxide 50 mg	↓COWS 28 → 3 *‘*Resolved within 4 hours’	NR
Lintzeris *et al*. [58]	Case description: 1 case within a RCT	Outpatient clinic	‘Patient reporting a transient increase in withdrawal symptoms in the hours immediately after dosing’	Heroin dependent	BPN 6 mg s/l	1	NR	NR	Australia	Clonidine Diazepam	NR	Not retained
Clark *et al*. [59]	Case report	Home → outpatient clinic	‘Agitation, nausea, sweating and abdominal cramps within an hour of ingestion’	Heroin use	Illicit BPN 40 mg s/l	1	0:1	35	Australia	BPN 24 mg s/l +BPN 16–24 mg s/l +BPN 16 mg s/l +Methadone	‘Continued to experience persistent agitation, poor sleep, abdominal cramps, diarrhoea and sweating’	NR
Banys *et al*. [60]	Case description: 1 case within an open trial	Outpatient clinic	↑Himmelsbach score 2 → 21	Methadone 50 mg PO od	BPN 0.6 mg s/l	1	0:1	66	USA	Methadone 50 mg Clonidine 0.2 mg Diazepam 10 mg	‘Withdrawal symptoms were mostly relieved in 6 hours’	Not retained

Notes: COWS is an 11 item clinician‐rated scale with scores 0–47; higher scores indicate more severe withdrawal symptoms [18].

Abbreviations: μg, microgram; bd, twice per day; BPN, buprenorphine; BPN/NX, buprenorphine/naloxone; COWS, Clinical Opiate Withdrawal Scale; ED, emergency department; F, female; IM, intramuscular; IN, intranasal; IV, intravenous; LAIB, long‐acting injectable buprenorphine; M, male; mg, milligram; NR, not reported; od, once per day; PO, per os (oral); s/l sublingual; tds, three times per day.

### Data extraction

Two authors independently extracted data from all eligible studies using a standardised data extraction spreadsheet that can be found in Table S2. In the case of incomplete reporting of data, we searched studies' online supplementary appendices and contacted authors as necessary. The main outcomes extracted were (a) any reported change in OWS [as assessed by any measure, e.g. via the use of a validated scale such as the Clinical Opiate Withdrawal Scale (COWS) or via narrative description] [18] and (b) the number of individuals reported as retained in buprenorphine treatment.

### Quality assessment

Where possible, outcome quality was assessed using the GRADE framework with risk of bias in included randomised controlled trials (RCTs) assessed using the Cochrane risk of bias assessment tool 2 (RoB‐2) [61] and in included observational studies using the applicable Newcastle Ottawa Scale (NOS) [62]. All evidence from case series and case reports was *a priori* deemed of very low quality. Two reviewers independently scored the quality of each study. Any discrepancy was resolved by discussion and where agreement could not be reached a third author was consulted.

### Statistical analysis

Where sufficient data were presented, we planned to combine outcomes in pairwise meta‐analysis. As high levels of heterogeneity were anticipated we planned to use a random effects model using Stata IC version 17, the significance level set at 0.05. Where only outcome descriptions were stated these were reported narratively. Ultimately, meta‐analysis was not possible because of lack of available data.

## RESULTS

The search generated 425 unique results and 26 additional references were identified from citation searching leading to a total of 451. We examined 142 full texts and included 43 studies. Ninety‐nine studies were excluded, the most common reason being that the study did not report a pharmacological treatment strategy for BPOW (*n* = 47). Full reasons for each study's exclusion can be found in Table S3 alongside the PRISMA diagram in Figure S2 describing study selection.

The 43 included studies comprised one pilot RCT and 42 uncontrolled observational studies. The observational studies comprised six case series, 22 case reports and 14 studies reporting descriptions of BPOW cases managed with pharmacological interventions described within wider studies (e.g. RCTs, cohort studies *etc*.). All observational studies reported only descriptive outcomes and as such meta‐analysis was not possible. All results are reported descriptively.

### Randomised evidence

The included pilot RCT was conducted in Iran within an emergency department (ED) setting and randomised a total of 40 individuals with BPOW. A summary is available in Table 1.

### Change in OWS

The included pilot RCT reported that, when compared to placebo plus conventional therapies (clonidine, diazepam and paracetamol) (*n* = 20), intravenous magnesium sulphate (MgSO_4_) plus conventional therapies (*n* = 20) significantly reduced mean COWS score at both 30 minutes [mean (SD); 14.4 (3.3) vs. 11.2 (2.9), *P* = 0.002] and 2 hours [11.3 (3.3) vs. 3.2 (1.6), *P* < 0.0001] post‐treatment. Forest plots are available in the online supplementary material as Figure S4.

### Number of individuals retained in buprenorphine treatment

The included pilot RCT did not report the number of individuals retained in buprenorphine treatment.

The Cochrane risk of bias assessment tool 2 (RoB2) can be found in the online supplementary material as Figure S3. The full GRADE clinical evidence profile can be seen in Table S4 with very serious risk of bias because of methodological limitations noted for both outcomes and the quality of mean COWS score at 30 minutes and 2 hours assessed overall as *very low* and *low*, respectively.

### Observational evidence

Included observational studies reported on a total of 97 individuals with BPOW managed with pharmacological interventions with each included study reporting on between one and 10 cases. Eighty‐five were treated in the United States, seven in Australia, four in Canada and one in the United Kingdom. Where reported, individual's ages ranged from 21 to 72 years with 25.5% (*n* = 13) reported to be female. The most commonly reported opioids, which had been used before precipitated withdrawal were fentanyl (*n* = 39, 43.3%), heroin (*n* = 27, 30.0%) and methadone (*n* = 24, 26.7%), with triggering doses of buprenorphine ranging between and 0.45 mg and 40 mg. The majority of individuals were reported to be managed either within EDs (*n* = 38, 43.7%) or hospital in‐patient settings (*n* = 25, 28.7%), with a minority managed in community settings (*n* = 24, 27.5%). Only five studies (11.9%) reported a formal definition for the diagnosis of BPOW. A summary of included observational studies, with outcomes reported verbatim, is available in Table 2.

### Pharmacological interventions

A total of 32 individual pharmacological agents were reportedly used to treat BPOW either as monotherapies or in combination. The three most common individual agents were additional doses of transmucosal buprenorphine (*n* = 74, 76.3%), clonidine (*n* = 30, 30.9%) and hydromorphone (*n* = 19, 19.6%). The six overall pharmacological treatment strategies used were (a) additional doses of buprenorphine as monotherapy (*n* = 15, 15.5%); (b) additional doses of buprenorphine in combination with symptomatic agents (e.g. clonidine, benzodiazepines *etc*.) (*n* = 43, 44.3%); (c) additional doses of buprenorphine in combination with both full opioid agonists (e.g. hydromorphone or methadone) and symptomatic agents (*n* = 16, 16.5%); (d) full opioid agonists in combination with symptomatic agents (*n* = 4, 4.1%); (e) full opioid agonists alone (*n* = 8, 8.2%); and (f) symptomatic agents alone (*n* = 11, 11.3%). Results stratified by these six treatment strategies can be found in Table S5.

### Change in OWS

The overall reported proportion of individuals experiencing a reduction or alleviation of OWS was 66.0% (*n* = 64). When the change in OWS was examined across the six different treatment strategies, there were comparatively higher proportions of individuals experiencing a reduction or alleviation of OWS only in strategies that included buprenorphine, i.e. strategy (a) additional doses of buprenorphine as monotherapy (*n* = 11, 73.3%); strategy (b) additional doses of buprenorphine in combination with symptomatic agents (*n* = 30, 69.8%); and strategy (c) additional doses of buprenorphine in combination with both full opioid agonists and symptomatic agents (*n* = 13, 81.3%).

### Number of individuals retained in buprenorphine treatment

The overall reported proportion of individuals retained in buprenorphine treatment was 49.5% (*n* = 48). When the number retained was examined across the six differing treatment strategies, there were comparatively higher proportions of individuals retained in buprenorphine treatment in strategy (b) additional doses of buprenorphine in combination with symptomatic agents (*n* = 26, 60.5%) and strategy (c) additional doses of buprenorphine in combination with both full opioid agonists and symptomatic agents (*n* = 10, 62.5%). All observational evidence was derived from case descriptions, case series and case reports and as such was of *very low* quality.

## DISCUSSION

Only *low* or *very low* quality evidence was available for any pharmacological intervention in the treatment of BPOW. Other than one pilot RCT, all other published empirical evidence was confined to uncontrolled descriptive case series and reports from 97 individuals. The currently available randomised evidence suggests that use of intravenous magnesium sulphate, in addition to symptomatic treatment with clonidine, paracetamol and diazepam, may significantly reduce OWS through N‐methyl‐D‐aspartate receptor antagonism when compared to symptomatic treatment alone. The currently available observational evidence suggests that treatment strategies that include additional doses of transmucosal buprenorphine may demonstrate higher rates of symptom control and treatment retention.

This study has several strengths and limitations. We implemented robust methods to conduct the review, using a broad search strategy and a pre‐defined protocol to capture evidence from studies of any design. There is, however, an overall paucity of evidence on which to base recommendations, with a total of only 137 treated cases reported in the extant literature. Of these, only a quarter of cases were female, almost two‐thirds were treated in the United States and less than a fifth were managed in community settings, substantially limiting the generalisability of any claims (e.g. the use of intravenous agents or medications requiring substantial monitoring are unlikely to be considered appropriate for use outside hospital environments). Only five studies documented a formal diagnostic definition, which may make differentiating BPOW from other causes of withdrawal symptoms, such as buprenorphine under dosing, difficult [6]. The composite observational evidence is also subject to potential selection bias, given the propensity to publish cases descriptions in which treatment has been deemed successful [63]. Nevertheless, this represents the first attempt to synthesise the totality of the evidence base on pharmacological management of BPOW adding to the literature to demonstrate the observational superiority of regimens in which additional doses of buprenorphine are administered.

When examined in the wider context of precipitated withdrawal, which has been triggered by opioid antagonists, buprenorphine is not typically recommended as a first‐line treatment option, with full agonists and symptomatic agents often favoured for symptom control [64]. There is understandable reticence surrounding the use of additional doses of buprenorphine in precipitated withdrawal, among both people with OUD and practitioners, for fear of continued exacerbation of withdrawal symptoms. We hope this review provides some reassurance as to the relative success of strategies involving additional doses of buprenorphine in published cases. Although only one pilot RCT has been conducted in this area, potentially because of low incidence and the operational challenges of randomising individuals with distressing BPOW symptomatology [3, 6], its existence does demonstrate the ability to conduct experimental research in this area. Given increasingly novel induction regimens, including direct induction of people using full agonists onto long‐acting injectable buprenorphine formulations [65], well‐designed RCTs within this area should be prioritised. Future research should report formal diagnostic definitions of BPOW, use validated scales to assess withdrawal symptomatology and aim for equity with regards gender representation. Anecdotally, several centres are also now using ketamine in cases of refractory BPOW, this also could be potentially a fruitful avenue for future research. Although further studies are essential to support the results reported in the included pilot RCT, magnesium sulphate may potentially represent a useful adjunct to symptomatic treatment in settings where intravenous access is available and can be managed safely.

## CONCLUSION

When considering possible interventions, people with OUD and practitioners need to be aware the best available evidence for pharmacological treatment in BPOW is limited and largely of very low quality. Although this review demonstrates a clear need for more rigorous experimental studies in the management of BPOW, there are some promising avenues for future potential research based on the published literature. Given the limited *very low* quality evidence, treatment regimens that include magnesium sulphate and additional doses of transmucosal buprenorphine may appear the most salient current options and avenues for future exploration in the management of BPOW.

## AUTHOR CONTRIBUTIONS

**Emmert Roberts:** Conceptualization; investigation; funding acquisition; writing—original draft; methodology; software; formal analysis; project administration; resources. **Nicola Kalk:** Investigation; writing—review and editing; validation. **John Strang:** Investigation; validation; writing—review and editing.

## DECLARATION OF INTERESTS

All authors have completed the ICJME Unified Competing Interest form (available on request from the corresponding author) and declare: no support from any organisation for the submitted work; no financial relationships with organisations that might have an interest in the submitted work in the previous 3 years; and no other relationships or activities that could appear to have influenced the submitted work.

## Supporting information


**Figure S1.** Search strategy
**Figure S2.** PRISMA flow diagram describing study selection
**Figure S3.** Cochrane risk of bias assessment tool 2 (RoB‐2) for included controlled studies
**Figure S4.** Forest plots for mean Clinical Opiate Withdrawal Scale (COWS) score post‐treatment
**Table S1.** Study protocol
**Table S2.** Data extraction spreadsheet
**Table S3.** Excluded studies
**Table S4.** GRADE clinical evidence profile for mean Clinical Opiate Withdrawal Scale (COWS) score post‐treatment
**Table S5.** The change in individual Opioid Withdrawal Symptoms (OWS) and number of individuals retained in buprenorphine (BPN) treatment stratified by the six pharmacological treatment strategies reported in observational studies
**Table S6.** Preferred Reporting Items for Systematic Reviews and Meta‐Analyses (PRISMA) checklist

## Data Availability

The data that support the findings of this systemic review are available as published articles.
