# Supplementary material for: Pharmacological treatment strategies to manage precipitated withdrawal following the administration of buprenorphine in opioid use disorder: A systematic review
Source: Addiction. 2026 Jan 22;121(5):1083–99. doi: 10.1111/add.70334 (PMC13088937; doi:10.1111/add.70334)
Supplement: Supplementary file 1 — Figure S1. Search strategy Figure S2. PRISMA flow diagram describing study selection Figure S3. Cochrane risk of bias assessment tool 2 (RoB‐2) for included controlled studies Figure S4. Forest plots for mean Clinical Opiate Withdrawal Scale (COWS) score post‐treatment Table S1. Study protocol Table S2. Data extraction spreadsheet Table S3. Excluded studies Table S4. GRADE clinical evidence profile for mean Clinical Opiate Withdrawal Scale (COWS) score post‐treatment Table S5. The change in individual Opioid Withdrawal Symptoms (OWS) and number of individuals retained in buprenorphine (BPN) treatment stratified by the six pharmacological treatment strategies reported in observational studies Table S6. Preferred Reporting Items for Systematic Reviews and Meta‐Analyses (PRISMA) checklist [file ADD-121-1083-s001.docx]

**Online Supplementary Material**

Contents

Figures

S1. Search strategy

S2. PRISMA flow diagram describing study selection

S3. Cochrane risk of bias assessment tool 2 (RoB-2) for included controlled studies

S4. Forest plots for mean Clinical Opiate Withdrawal Scale (COWS) score post-treatment

Tables

S1. Study protocol

S2. Data extraction spreadsheet

S3. Excluded studies

S4. GRADE clinical evidence profile for mean Clinical Opiate Withdrawal Scale (COWS) score post-treatment

S5. The change in individual Opioid Withdrawal Symptoms (OWS) and number of individuals retained in buprenorphine (BPN) treatment stratified by the six pharmacological treatment strategies reported in observational studies

S6. Preferred Reporting Items for Systematic Reviews and Meta-Analyses (PRISMA) checklist

References

Figures

Figure S1: Search strategy

((precipitat*) AND (withdraw*) AND (buprenorphine OR subutex OR suboxone OR buvidal OR sublocade OR espranor)).mp

Figure S2: PRISMA flow diagram describing study selection

Records identified through database searching
(n = 425)

## Screening

## Included

## Eligibility

## Identification

Additional records identified through other sources
(n = 26)

Records after duplicates removed
(n = 451)

Records screened
(n = 451)

Records excluded
(n = 309)

Full-text articles assessed for eligibility
(n = 142)

Full-text articles excluded, with reasons*
(n = 99)

*Please see table S3 in online supplementary material for full reasons for all excluded articles

Studies included
(n = 43)

Figure S3: Cochrane risk of bias assessment tool 2 (RoB 2) for included randomised studies


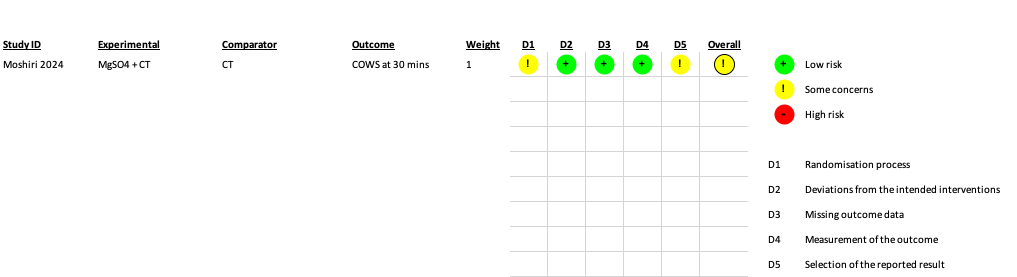


Figure S4: Forest plots for mean Clinical Opiate Withdrawal Scale (COWS) score post-treatment (1)

1. 30 minutes post-treatment

1. 2 hours post-treatment

Tables

Table S1: Protocol

| Population | Any adult experiencing buprenorphine precipitated opioid withdrawal (BPOW) (aged ≥ 18 years) |
| --- | --- |
| Intervention | Any pharmacological treatment strategy |
| Comparison | Any or none |
| Outcomes | Any measurement or description of the change in opioid withdrawal symptoms following administration of pharmacotherapy (as assessed by any measure e.g., the use of a validated scale such as the Clinical Opiate Withdrawal Scale (COWS) (1) or a narrative description)  Any measurement or description of the number of individuals retained in buprenorphine treatment. |
| Exclusions | Any study where a pharmacological intervention was not being used as a treatment for BPOW |
| Study Design | Any |

Table S2: Data extraction spreadsheet

| Study ID | Study Design | Population | | | | | | | | Intervention/s | Comparison/s | Outcomes | |
| --- | --- | --- | --- | --- | --- | --- | --- | --- | --- | --- | --- | --- | --- |
|  |  |  |  |  |  |  |  |  |  |  |  | Change in Opioid  Withdrawal Symptoms | Retained in BPN Treatment? (n) |
|  |  | Setting | Definition/ Reported symptoms of Precipitated Withdrawal | Baseline  Opioid Use | Administered BPN  Formulation/Dose | n | F:M | Age (years) | Country |  |  |  |  |

Table S3: Excluded studies

| Citation | Reason for exclusion |
| --- | --- |
| TREMONTI C., BLOGG J., JAMSHIDI N., HARJANTO R., MILES N., ISMAY C. et al. Methadone-Buprenorphine Transfers Using Low Dosing of Buprenorphine: An Open-label, Nonrandomized Clinical Trial, Journal of Addiction Medicine 2025: 19: 75-82. | Exclude: Does not report treatment strategies for BPOW; All references checked for includable citations |
| SUEN L. W., CHIANG A. Y., JONES B. L. H., SORAN C. S., GEIER M., SNYDER H. R. et al. Outpatient Low-Dose Initiation of Buprenorphine for People Using Fentanyl, JAMA Network Open 2025: 8: e2456253. | Exclude: Does not report treatment strategies for BPOW |
| SHEN M. R., CAMPBELL D. E., KOPCZYNSKI A., MADDAMS S., ROSENBLUM N., NIGAM K. et al. Ketamine in treating opioid use disorder and opioid withdrawal: a scoping review, Frontiers in Psychiatry 2025: 16(no pagination). | Exclude: Review article all references checked for includable studies |
| NAREN T., MEMBERY D., MACCARTNEY P., D'CUNHA R., NIELSEN S., GARRY S. Feasiblity of Direct Induction Onto Long-Acting Injectable Buprenorphine, Ssrn 2025: 14. | Exclude: Does not report treatment strategies for BPOW. reports BPOW cases were “treated symptomatically” |
| JACOBSON K. M., PITTIGLIO M., EID S., VASSALLO A. Analysis of a High-Dose Buprenorphine Induction Protocol for the Treatment of Opioid Use Disorder in the Emergency Department, American Journal of Health-System Pharmacy 2025: 82(Supplement 1): S1592. | Exclude: Conference abstract does not report treatment strategies for BPOW |
| GURLEY A. L., LACOCQUE J., MERCER M. P., MASON M., WIEBERS J., LARA V. et al. Prehospital Buprenorphine in Treating Symptoms of Opioid Withdrawal - A Descriptive Review of the First 131 Cases in San Francisco, CA, Prehospital Emergency Care 2025: 29: 435-440. | Exclude: Does not report treatment strategies for BPOW “one patient was treated for suspected buprenorphine-precipitated withdrawal” |
| GRENIER K. A., SANDERSON C. S., PAOLETTA G., ADAMS K. K. Low-dose Buprenorphine Initiation in Pregnancy: A Systematic Review, Journal of Addiction Medicine 2025: (no pagination). | Exclude: Review article all references checked for inclusion |
| GREGORY C., YADAV K., LINDERS J., SIKORA L., EAGLES D. Incidence of buprenorphine-precipitated opioid withdrawal in adults with opioid use disorder: A systematic review, Addiction 2025: 120: 7-20. | Exclude: Review article reporting incidence of BPOW; Nil treatment strategies for BPOW reported. All citations checked for inclusion |
| GLENN M. J., ERSTAD B. L. Challenges with current diagnosis and treatment strategies for precipitated opioid withdrawal in the emergency department and the role of the pharmacist, American Journal of Health-System Pharmacy 2025: 82: 60-64. | Exclude: Review article all references checked for inclusion |
| CHIU I., DONEGAN K., GINGOLD D., POREMBA M., STRYCKMAN B., WILKERSON R. 556 Precipitated Withdrawal Following Emergency Department-Initiated Buprenorphine, Annals of Emergency Medicine 2025: 86(3 Supplement 1): S238-S239. | Exclude: Does not report treatment strategies for BPOW |
| ARMOUR R., NIELSEN S., BUXTON J. A., BOLSTER J., HAN M. X., ROSS L. Initiation of buprenorphine in the emergency department or emergency out-of-hospital setting: A mixed-methods systematic review, American Journal of Emergency Medicine 2025: 88: 12-22. | Exclude: Review article all references checked for inclusion |
| WONG S., FABIANO N., WEBBER D., KLEINMAN R. A. High-Dose Buprenorphine Initiation: A Scoping Review, Journal of Addiction Medicine 2024: 18: 349-359. | Exclude: Review article all references checked for inclusion |
| THAKRAR A. P., CHRISTINE P. J., SIAW-ASAMOAH A., SPADARO A., FAUDE S., SNIDER C. K. et al. Buprenorphine-Precipitated Withdrawal Among Hospitalized Patients Using Fentanyl, JAMA Network Open 2024: 7: e2435895. | Exclude: Does not report treatment strategies for BPOW |
| STRAND A., GORDON B., WILKINSON J. Current Challenges of Dosing, Management, And Precipitated Withdrawal with Suboxone For Opiate Use Disorder, Western Journal of Emergency Medicine 2024: 25(2.1): S29. | Exclude: Conference abstract reporting presentation of management of BPOW no empirical data presented |
| SOARES W. E., SCHOENFELD E., FRIEDMANN P. D. Precipitated Withdrawal in the Era of Street Fentanyl - The Important Thing Is to Not Stop Questioning, JAMA Network Open 2024: 7(9): e2435857. | Exclude: Commentary does not report treatment strategies for BPOW |
| REDDY S., MARTIN C. E. Low-dose buprenorphine initiation during pregnancy: a case report, AJOG Global Reports 2024: 4(1) (no pagination). | Exclude: Describes one case with BPOW but patient did not receive any pharmacological treatment and was re inducted. |
| PRAEGER V. C., FREI M. Y., PHAM D., PRAEGER A. J., LUBMAN D. I., ARUNOGIRI S. Rotation from methadone to buprenorphine using a micro-dosing regime in patients with opioid use disorder and serious mental illness: A case series, Drug Alcohol Rev 2024: 43: 1829-1834. | Exclude: Describes a patient experiencing BPOW however “patient ceased the transition process after this” no treatment strategies reported. |
| JONES B. L. H., GEIER M., NEUHAUS J., COFFIN P. O., SNYDER H. R., SORAN C. S. et al. Withdrawal during outpatient low dose buprenorphine initiation in people who use fentanyl: a retrospective cohort study, Harm Reduction Journal 2024: 21: 80. | Exclude: Does not report treatment strategies for BPOW. All references checked for inclusion. |
| ISENBERG R. Opioid overdose after extended-release buprenorphine injection: A case report, The American Journal of Drug and Alcohol Abuse 2024: 50: 123-124. | Exclude: Does not report treatment strategies for BPOW |
| HUGHES T., NASSER N., MITRA A. Overview of best practices for buprenorphine initiation in the emergency department, International Journal of Emergency Medicine 2024: 17(1) (no pagination). | Exclude: Review article presents guidelines for pharmacological management of BPOW with no empirical data, only reference cross checked for inclusion. |
| HAYES B. T., LI P., NIENALTOW T., TORRES-LOCKHART K., KHALID L., FOX A. D. Low-dose buprenorphine initiation and treatment continuation among hospitalized patients with opioid dependence: A retrospective cohort study, Journal of Substance Use and Addiction Treatment 2024: 158: 209261. | Exclude: Does not report treatment strategies for BPOW. All references checked for inclusion |
| GRANDE L. A., HUTCH T., JACK K., MIRONOV W., IWUOHA J., MUY-RIVERA M. et al. Ketamine-assisted buprenorphine initiation: a pilot case series, Addiction Science & Clinical Practice 2024: 19: 60. | Exclude: Does not report result separately for people experiencing BPOW and those who are using ketamine prophylactically to avoid withdrawal symptoms |
| ERSTAD B. L., GLENN M. J. Considerations and limitations of buprenorphine prescribing for opioid use disorder in the intensive care unit setting: A narrative review, American Journal of Health-System Pharmacy 2024: 81: 171-182. | Exclude: Review article all references checked for inclusion |
| D'ONOFRIO G., HERRING A. A., PERRONE J., HAWK K., SAMUELS E. A., COWAN E. et al. Extended-Release 7-Day Injectable Buprenorphine for Patients With Minimal to Mild Opioid Withdrawal, JAMA Network Open 2024: 7: e2420702. | Exclude: Does not report treatment if patient with BPOW: Reports ancillary medications for those without BPOW but not treatment strategies for n=7 with precipitated withdrawal |
| ARNOUK S., WUNDERLICH J. R., SIDELNIK S. A. Evaluation of Low-dose Buprenorphine Initiation With Buprenorphine Buccal Films in Hospitalized Patients: A Retrospective Cohort Study, Journal of Addiction Medicine 2024: 18: 42-47. | Exclude: Does not report treatment strategies for BPOW |
| WUNSCH C., WIGHTMAN R., PRATTY C., JACKA B., HALLOWELL B. D., CLARK S. et al. Thirty-day Treatment Continuation After Audio-only Buprenorphine Telehealth Initiation, Journal of Addiction Medicine 2023: 17: 206-209. | Exclude: Does not report treatment strategies for BPOW |
| SULAKVELIDZE N., RONAN C., PETERKIN A. F., WEINSTEIN Z. M. Efficacy of Low-Dose Versus Traditional Buprenorphine Induction in the Hospital: A Quantitative and Qualitative Study, American Journal of Therapeutics 2023: 30: e1-e9. | Exclude: Does not report treatment strategies for BPOW |
| SNYDER H., CHAU B., KALMIN M. M., SPEENER M., CAMPBELL A., MOULIN A. et al. High-dose buprenorphine initiation in the emergency department among patients using fentanyl and other opioids, JAMA network open 2023: 6: e231572-e231572. | Exclude: Does not report treatment strategies for BPOW |
| SCHULT R. F., MAYNARD K. M., CORVELLI J. M., RAPPAPORT S., MCKINNEY B., CLARKSON T. et al. Low-dose Initiation of Buprenorphine in Hospitalized Patients on Full Agonist Opioid Therapy: A Retrospective Observational Study, Journal of Addiction Medicine 2023: 17: 685-690. | Exclude: reports two cases of BPOW: Does not report treatment for BPOW only medications administered within one hour of buprenorphine dose that triggered onset of BPOW. |
| NOEL M., ABBS E., SUEN L., SAMUEL L., DOBBINS S., GEIER M. et al. The Howard Street Method: A Community Pharmacy-led Low Dose Overlap Buprenorphine Initiation Protocol for Individuals Using Fentanyl, Journal of Addiction Medicine 2023: 17: e255-e261. | Exclude: Does not report treatment strategies for BPOW |
| LYNCH D., CHITTY L., JOHNSON B., HOEFNAGEL A. L. Suspected Buprenorphine-Precipitated Opioid Withdrawal following Intercourse: A Case Report, J Pain Pall Care Pharmacother 2023: 37: 314-316. | Exclude: Does not report treatment strategies for BPOW |
| KILARU A., PERRONE J., LOWENSTEIN M., NELSON L., SPADARO A., FAUDE S. et al. 302 Incidence of Buprenorphine-Precipitated Withdrawal in Emergency Department Patients With Opioid Use Disorder in Philadelphia, Annals of Emergency Medicine 2023: 82(4 Supplement): S132. | Exclude: Does not report treatment strategies for BPOW |
| JAIN L., MORRISROE K., MODESTO-LOWE V. To use or not to use buprenorphine for illegally manufactured fentanyl, Family Practice 2023: 40: 428-430. | Exclude: Commentary does not report on empirical cases of BPOW |
| HERN H. G., LARA V., GOLDSTEIN D., KALMIN M., KIDANE S., SHOPTAW S. et al. Prehospital Buprenorphine Treatment for Opioid Use Disorder by Paramedics: First Year Results of the EMS Buprenorphine Use Pilot, Prehospital Emergency Care 2023: 27: 334-342. | Exclude: None experienced precipitated withdrawal |
| EDINOFF A. N., FAHMY O. H., SPILLERS N. J., ZAHERI A. R., JACKSON E. D., DE WITT A. J. et al. Low-Dose Initiation of Buprenorphine: A Narrative Review, Curr Pain Headache Rep 2023: 27: 175-181. | Exclude: Review article reporting on induction does not report treatment strategies for BPOW |
| D'ONOFRIO G., HAWK K. F., PERRONE J., WALSH S. L., LOFWALL M. R., FIELLIN D. A. et al. Incidence of Precipitated Withdrawal During a Multisite Emergency Department-Initiated Buprenorphine Clinical Trial in the Era of Fentanyl, JAMA Network Open 2023: 6: e236108. | Exclude: Does not report treatment strategies for BPOW |
| BIRD H. E., HUHN A. S., DUNN K. E. Fentanyl Absorption, Distribution, Metabolism, and Excretion: Narrative Review and Clinical Significance Related to Illicitly Manufactured Fentanyl, Journal of Addiction Medicine 2023: 17: 503-508. | Exclude: Review article all references checked for inclusion |
| ADKINS A. Q., GOLDFARB D. A., BARTON D., KUKOWSKI M., PIZON A. F. Pumping the Brakes on Precipitated Withdrawal with a Transdermal Buprenorphine Patch, Journal of Medical Toxicology 2023: 19(2): 133. | Exclude: Does not report treatment strategies for BPOW |
| VARSHNEYA N. B., THAKRAR A. P., HOBELMANN J. G., DUNN K. E., HUHN A. S. Evidence of Buprenorphine-precipitated Withdrawal in Persons Who Use Fentanyl, Journal of Addiction Medicine 2022: 16: e265-e268. | Exclude: Cross sectional survey of BPOW symptoms in people using fentanyl. Does not report treatment strategies for BPOW |
| TOFIGHI B., MCNEELY J., YANG J., THOMAS A., SCHATZ D., REED T. et al. Outcomes of a NYC Public Hospital System Low-Threshold Tele-Buprenorphine Bridge Clinic at 1 Year, Substance Use & Misuse 2022: 57: 1337-1340. | Exclude: Does not report treatment strategies for BPOW |
| SPADARO A., LONG B., KOYFMAN A., PERRONE J. Buprenorphine precipitated opioid withdrawal: Prevention and management in the ED setting, American Journal of Emergency Medicine 2022: 58: 22-26. | Exclude: Review article all references checked for includable studies |
| SPADARO A., FAUDE S., LOWENSTEIN M., THAKRAR A., DELGADO M., PERRONE J. et al. 282 Buprenorphine-Precipitated Opioid Withdrawal in the Emergency Department: A Case Series, Annals of Emergency Medicine 2022: 80(4 Supplement): S122. | Exclude: Conference abstract of included study Spadaro 2023 |
| MALOUF C. A., LAI A., SINGHVI A. A Stressful Withdrawal: Buprenorphine-Induced Takotsubo Cardiomyopathy, Journal of the American College of Cardiology 2022: 79(9 Supplement): 2234. | Exclude: Does not report treatment strategies for BPOW |
| LINTZERIS N., MANKABADY B., ROJAS-FERNANDEZ C., AMICK H. Strategies for Transfer From Methadone to Buprenorphine for Treatment of Opioid Use Disorders and Associated Outcomes: A Systematic Review, Journal of Addiction Medicine 2022: 16: 143-151. | Exclude: Review article all included studies reporting on BPOW checked for inclusion |
| KOLOD E. B., SHERMAN J., RIAZI F., MAR Y. Collaboration Is Key: Buprenorphine Microinduction with Moderate Dose Methadone in a World Fraught with Fentanyl, Journal of General Internal Medicine 2022: 37(Supplement 2): S359. | Exclude: Does not report treatment strategies for BPOW |
| KARAVOLIS Z. A., ROY P. J. Adapting low-dose buprenorphine induction to meet patient needs: A pilot study, Drug and Alcohol Dependence Reports 2022: 5(no pagination). | Exclude: Does not report treatment strategies for BPOW |
| HAILOZIAN C., LUFTIG J., LIANG A., OUTHAY M., ULLAL M., ANDERSON E. S. et al. Synergistic Effect of Ketamine and Buprenorphine Observed in the Treatment of Buprenorphine Precipitated Opioid Withdrawal in a Patient With Fentanyl Use, Journal of Addiction Medicine 2022: 16: 483-487. | Exclude: Case report of patient included in Heeney 2022 from same centre at same time with same treatment. Exclude to avoid duplication. |
| FORAN A., TREMONTI C., BALGOBIND V., SAKR H., MURNION B. Transferring patients from high-dose methadone to buprenorphine: A retrospective case series, Journal of Opioid Management 2022: 18(6): 557-566. | Exclude: Does not report treatment strategies for BPOW |
| DAVID M. S., JONES J., LAURIELLO A., NNAKE I., PLAZAS MONTANA M., LASKO K. et al. Converting adults with sickle cell disease from full agonist opioids to buprenorphine: A reliable method with safety and early evidence of reduced acute care utilization, American Journal of Hematology 2022: 97: 1435-1442. | Exclude: Describes one case of protracted withdrawal not BPOW. |
| COMSTOCK G., TRUSZCZYNSKI N., HOPPE J. 343<sup>EMF</sup> Identifying and Addressing Barriers to Emergency Department Buprenorphine Use Across a Health Care System, Annals of Emergency Medicine 2022: 80(4 Supplement): S146. | Exclude: Conference abstract reporting the creation of summery of management for BPOW but no empirical data or summery presented |
| BUTTON D., HARTLEY J., ROBBINS J., LEVANDER X. A., SMITH N. J., ENGLANDER H. Low-dose Buprenorphine Initiation in Hospitalized Adults With Opioid Use Disorder: A Retrospective Cohort Analysis, Journal of Addiction Medicine 2022: 16: e105-e111. | Exclude: Does not report treatment strategies for BPOW |
| BROGDON H., FACER K. L., COX E. J., CARLSON R. H., JR., WURZEL J. F., 3RD. Rapid Transition to Buprenorphine in a Patient With Methadone-Related QTc Interval Prolongation, Journal of Addiction Medicine 2022: 16: 488-491. | Exclude: Describes a case of naltrexone precipitated withdrawal |
| BAUMGARTNER K., SALMO E., LISS D., DEVGUN J., MULLINS M., GALATI B. et al. Transdermal buprenorphine for in-hospital transition from full agonist opioids to sublingual buprenorphine: a retrospective observational cohort study, Clinical Toxicology: The Official Journal of the American Academy of Clinical Toxicology & European Association of Poisons Centres & Clinical Toxicologists 2022: 60: 688-693. | Exclude: Reports induction strategies not treatment of BPOW |
| TAY WEE TECK J., BALDACCHINO A., GIBSON L., LAFFERTY C. Using Microdosing to Induct Patients Into a Long-Acting Injectable Buprenorphine Depot Medication in Low Threshold Community Settings: A Case Study, Frontiers in Pharmacology 2021: 12(no pagination). | Exclude: Does not describe cases of BPOW |
| SOYKA M. Transition From Full Mu Opioid Agonists to Buprenorphine in Opioid Dependent Patients-A Critical Review, Frontiers in Pharmacology 2021: 12(no pagination). | Exclude: Reviews induction strategies does not discuss treatment strategies for BPOW. |
| POWELL V. D., ROSENBERG J. M., YAGANTI A., GARPESTAD C., LAGISETTY P., SHANNON C. et al. Evaluation of Buprenorphine Rotation in Patients Receiving Long-term Opioids for Chronic Pain: A Systematic Review, JAMA Network Open 2021: 4: e2124152. | Exclude: Does not report treatment strategies for BPOW |
| MOE J., O'SULLIVAN F., HOHL C. M., DOYLE-WATERS M. M., RONSLEY C., CHO R. et al. Short communication: Systematic review on effectiveness of micro-induction approaches to buprenorphine initiation, Addictive Behaviors 2021: 114: 106740. | Exclude: Review article reports on three cases/studies of BPOW but does not provide referenced nil treatment strategies reported. |
| KELLY J. C., RAGHURAMAN N., STOUT M. J., RUSSELL S., PEREZ M., NAZEER S. et al. Home induction of buprenorphine for treatment of opioid use disorder in pregnancy, Obstet Gynecol 2021: 138: 655-659. | Exclude: Does not report treatment strategies for BPOW |
| JACKSON P. 66.2 the Art of the Start: Clinical Readiness for Successful Initiation of Buprenorphine in Adolescents and Young Adults, Journal of the American Academy of Child and Adolescent Psychiatry 2021: 60(10 Supplement): S97-S98. | Exclude: Conference abstract reporting that clinical management strategies will be presented. |
| CARROLL G. G., WASSERMAN D. D., SHAH A. A., SALZMAN M. S., BASTON K. E., ROHRBACH R. A. et al. Buprenorphine field initiation of ReScue treatment by emergency medical services (Bupe FIRST EMS): a case series, Prehospital Emergency Care 2021: 25: 289-293. | Exclude: Does not report any cases of BPOW |
| AHMED S., BHIVANDKAR S., LONERGAN B. B., SUZUKI J. Microinduction of Buprenorphine/Naloxone: A Review of the Literature, American Journal on Addictions 2021: 30: 305-315. | Exclude: Review article all references checked for includable studies |
| ZAMANI N., BUCKLEY N. A., HASSANIAN-MOGHADDAM H. Buprenorphine to reverse respiratory depression from methadone overdose in opioid-dependent patients: a prospective randomized trial, Crit Care 2020: 24: 44. | Exclude: Does not report treatment strategies for BPOW |
| STRAYER R. J., HAWK K., HAYES B. D., HERRING A. A., KETCHAM E., LAPIETRA A. M. et al. Management of opioid use disorder in the emergency department: a white paper prepared for the American Academy of Emergency Medicine, The Journal of emergency medicine 2020: 58: 522-546. | Exclude: Guideline for management all references checked for potential inclusion |
| KUNZLER N. M., WIGHTMAN R. S., NELSON L. S. Opioid Withdrawal Precipitated by Long-Acting Antagonists, Journal of Emergency Medicine 2020: 58: 245-253. | Exclude: Reports withdrawal precipitated by opioid antagonists not buprenorphine |
| EDWARDS F. J., WICELINSKI R., GALLAGHER N., MCKINZIE A., WHITE R., DOMINGOS A. Treating Opioid Withdrawal With Buprenorphine in a Community Hospital Emergency Department: An Outreach Program, Annals of Emergency Medicine 2020: 75: 49-56. | Exclude: No instances of precipitated withdrawal observed |
| BRAR J. K., BROYAN V. R., ALLGAIER J. T., NYE L., SAXON A. J. Long-term Buprenorphine Treatment for Loperamide Use Disorder: A Case Series, Journal of Addiction Medicine 2020: 14: e378-e381. | Exclude: Reports two cases of BPOW but nil reported treatment strategies |
| BHATIA G., SARKAR S. Sublingual buprenorphine-naloxone precipitated withdrawal-A case report with review of literature and clinical considerations, Asian Journal of Psychiatry 2020: 53: 102121. | Exclude: Not BPOW - Case of precipitated withdrawal when person on stable dosage of buprenorphine given buprenorphine-naloxone combination - only doses containing naloxone triggered withdrawal symptoms - Not BPOW |
| HERRING A. A., PERRONE J., NELSON L. S. Managing opioid withdrawal in the emergency department with buprenorphine, Annals of Emergency Medicine 2019: 73: 481-487. | Exclude: Clinical algorithm recommending buprenorphine to treat BPOW. All references checked for potential inclusion |
| CISEWSKI D. H., SANTOS C., KOYFMAN A., LONG B. Approach to buprenorphine use for opioid withdrawal treatment in the emergency setting, American Journal of Emergency Medicine 2019: 37: 143-150. | Exclude: Makes treatment recommendation for clonidine and lorazepam: No empirical data all references checked for potential inclusion. |
| ABDELRAHMAN A., BORDEN M., GHAMLOUSH M. A. Agitated delirium in an intravenous drug user: Don't jump to conclusions, American Journal of Respiratory and Critical Care Medicine Conference: American Thoracic Society International Conference, ATS 2018: 197. | Exclude: Reports management of precipitated serotonin syndrome not BPOW |
| SURMAITIS R., KHALID M., VEARRIER D., GREENBERG M. I. Takotsubo cardiomyopathy associated with buprenorphine precipitated withdrawal, Clinical Toxicology 2017: 55(7): 752-753. | Exclude: Conference abstract of Surmaitis 2018 |
| BHATRAJU E. P., GROSSMAN E., TOFIGHI B., MCNEELY J., DIROCCO D., FLANNERY M. et al. Public sector low threshold office-based buprenorphine treatment: outcomes at year 7, Addiction Science & Clinical Practice 2017: 12: 7. | Exclude: Does not report treatment strategies for BPOW |
| WEBSTER L. R., SMITH M. D., UNAL C., FINN A. Low-dose naloxone provides an abuse-deterrent effect to buprenorphine, Journal of Pain Research 2015: 8: 791-798. | Exclude: Reports use of “rescue medication” to treat BPOW with no further elaboration |
| SUZUKI J., DEVIDO J., KALRA I., MITTAL L., SHAH S., ZINSER J. et al. Initiating buprenorphine treatment for hospitalized patients with opioid dependence: a case series, The American journal on addictions 2015: 24: 10-14. | Exclude: Does not report treatment strategies for BPOW |
| SUMNER M., WEBSTER L. R., HJELMSTR'OM P., GUNDERSON E. W. Advanced-Formulation Sublingual Buprenorphine/Naloxone Tablet is Effective in Induction Treatment of Opioid-Dependent Adults Track: Pharmacotherapy, Journal of Addiction Medicine 2015: 9(4): E8-E9. | Exclude: Conference abstract does not report treatment strategies for BPOW |
| HILL D., CONROY S. Case note review - Transfer of patient to buprenorphine from daily doses of methadone greater than 30mg, Heroin Addiction and Related Clinical Problems 2014: 16(1): 55-62. | Exclude: Reports transfer procedure in five patients does not report treatment strategies for confirmed BPOW |
| CONROY S., HILL D. Transfer to buprenorphine from daily doses of methadone greater than 30 mg—initial review of transfers, Heroin Addict Relat Clin Probl 2013: 15: 19-28. | Exclude: Does not report treatment strategies for BPOW |
| ROSENBLUM A., CRUCIANI R. A., STRAIN E. C., CLELAND C. M., JOSEPH H., MAGURA S. et al. Sublingual buprenorphine/naloxone for chronic pain in at-risk patients: development and pilot test of a clinical protocol, Journal of Opioid Management 2012: 8: 369-382. | Exclude: Does not report treatment strategies for potential BPOW |
| NIELSEN S., HILLHOUSE M., MOONEY L., FAHEY J., LING W. Comparing buprenorphine induction experience with heroin and prescription opioid users, Journal of substance abuse treatment 2012: 43: 285-290. | Exclude: Does not report treatment strategies for BPOW |
| OMOIGUI S., HASHMAT F., BERNARDO Z. Use of ketamine in ameliorating opioid withdrawal symptoms during an induction phase of buprenorphine, The Open Pain Journal 2011: 4. | Exclude: Does not report cases of BPOW |
| KRAUS M. L., ALFORD D. P., KOTZ M. M., LEVOUNIS P., MANDELL T. W., MEYER M. et al. Statement of the American Society of Addiction Medicine Consensus Panel on the use of buprenorphine in office-based treatment of opioid addiction, Journal of addiction medicine 2011: 5: 254-263. | Exclude: Treatment recommendations buprenorphine plus symptomatics. All references checked for potential inclusion |
| DOOLITTLE B., BECKER W. A case series of buprenorphine/naloxone treatment in a primary care practice, Substance Abuse 2011: 32: 262-265. | Exclude: Does not report treatment strategies for BPOW |
| GUNDERSON E. W., WANG X.-Q., FIELLIN D. A., BRYAN B., LEVIN F. R. Unobserved versus observed office buprenorphine/naloxone induction: a pilot randomized clinical trial, Addictive behaviors 2010: 35: 537-540. | Exclude: Does not report treatment strategies for BPOW |
| COULSON J., ROUTLEDGE P. A. Adverse reactions to drug withdrawal, Adverse Drug Reaction Bulletin 2008: 967-970. | Exclude: Does not discuss BPOW |
| ROSADO J., WALSH S. L., BIGELOW G. E., STRAIN E. C. Sublingual buprenorphine/naloxone precipitated withdrawal in subjects maintained on 100mg of daily methadone, Drug & Alcohol Dependence 2007: 90: 261-269. | Exclude: Reports prevention strategies not treatment strategies for BPOW. |
| BERG M., IDREES U., DING R., NESBIT S., LIANG H., MCCARTHY M. Evaluation of the use of buprenorphine for opioid withdrawal in an emergency department, Drug and alcohol dependence 2007: 86: 239-244. | Exclude: No cases of BPOW reported |
| JOVAIšA T., LAURINėNAS G., VOSYLIUS S., ŠIPYLAITė J., BADARAS R., IVAšKEVIčIUS J. Effects of ketamine on precipitated opiate withdrawal, Medicina (Kaunas) 2006: 42: 625-634. | Exclude: Evaluation of ketamine to prevent symptoms of opioid antagonist precipitated withdrawal |
| CLARK N. C. Expanding the Options for the Treatment of Heroin Dependence with Oral Supervised Opioid Substitution Therapy: LAAM, Buprenorphine and Slow Release Oral Morphine as Alternatives to Methadone: University of Melbourne, Department of Medicine, St Vincent's Hospital; 2006. | Exclude: Thesis reports that in cases of BPOW “non opiate medication was used to settle withdrawal symptoms |
| UMBRICHT A., HUESTIS M. A., CONE E. J., PRESTON K. L. Effects of high-dose intravenous buprenorphine in experienced opioid abusers, Journal of clinical psychopharmacology 2004: 24: 479-487. | Exclude: Experimental study of IV buprenorphine in non-dependent opioid users |
| GREENWALD M. K., SCHUH K. J., STINE S. M. Transferring methadone-maintained outpatients to the buprenorphine sublingual tablet: a preliminary study, American Journal on Addictions 2003: 12: 365-374. | Exclude: Does not report treatment strategies for BPOW |
| BREEN C. L., HARRIS S. J., LINTZERIS N., MATTICK R. P., HAWKEN L., BELL J. et al. Cessation of methadone maintenance treatment using buprenorphine: Transfer from methadone to buprenorphine and subsequent buprenorphine reductions, Drug and Alcohol Dependence 2003: 71(1): 49-55. | Exclude: Does not report pharmacological treatment strategies to manage BPOW. Reports some people with BPOW used heroin or illicit methadone but nil report of outcomes. |
| JACOBS E. A., BICKEL W. K. Precipitated withdrawal in an opioid-dependent outpatient receiving alternate-day buprenorphine dosing, Addiction 1999: 94: 140-141. | Exclude: Does not report treatment strategies for BPOW |
| BOUCHEZ J., BEAUVERIE P., TOUZEAU D. Substitution with buprenorphine in methadone- and morphine sulfate-dependent patients. Preliminary results, European Addiction Research 1998: 4 Suppl 1: 8-12. | Exclude: Does not report treatment strategies for BPOW |
| LEVIN F. R., FISCHMAN M. W., CONNERNEY I., FOLTIN R. W. A protocol to switch high-dose, methadone-maintained subjects to buprenorphine, American Journal on Addictions 1997: 6: 105-116. | Exclude: Reports use of ancillary medication during methadone to buprenorphine switch but no specific discussion of which subjects experienced BPOW |
| MENDELSON J., JONES R. T., FERNANDEZ I., WELM S., MELBY A. K., BAGGOTT M. J. Buprenorphine and naloxone interactions in opiate-dependent volunteers, Clinical Pharmacology & Therapeutics 1996: 60: 105-114. | Exclude: Reports experimental naloxone precipitated withdrawal |
| WALSH S. L., JUNE H. L., SCHUH K. J., PRESTON K. L., BIGELOW G. E., STITZER M. L. Effects of buprenorphine and methadone in methadone-maintained subjects, Psychopharmacology 1995: 119: 268-276. | Exclude: Describes cases of BPOW but no pharmacological treatment strategy |
| STRAIN E. C., PRESTON K. L., LIEBSON I. A., BIGELOW G. E. Buprenorphine effects in methadone-maintained volunteers: effects at two hours after methadone, The Journal of pharmacology and experimental therapeutics 1995: 272: 628-638. | Exclude: Experimental study of precipitated withdrawal no treatment strategies reported |
| KOSTEN T. R., MORGAN C., KLEBER H. D. Treatment of heroin addicts using buprenorphine, American Journal of Drug & Alcohol Abuse 1991: 17: 119-128. | Exclude: Does not report treatment strategies for BPOW |
| LUKAS S. E., JASINSKI D. R., JOHNSON R. E. Electroencephalographic and behavioral correlates of buprenorphine administration, Clinical Pharmacology & Therapeutics 1984: 36: 127-132. | Exclude: Does not report treatment strategies for BPOW |

Table S4: GRADE clinical evidence profile for mean post-treatment Clinical Opiate Withdrawal Scale (COWS) (1)

| **Quality assessment** | | | | | | | **Summary of findings** | | | **Quality** |
| --- | --- | --- | --- | --- | --- | --- | --- | --- | --- | --- |
|  |  |  |  |  |  |  | **Intervention (n)**  **Mean (SD)** | **Control (n)**  **Mean (SD)** | **Effect** |  |
| **No of studies** | **Design** | **Risk of bias** | **Inconsistency** | **Indirectness** | **Imprecision** | **Other considerations** |  |  | **Mean Difference (MD) (95% CI)** |  |
| Mean Clinical Opiate Withdrawal Scale (COWS) Score at 30 minutes post-treatment | | | | | | | | | | |
| 1  Moshiri 2024 | Randomised trial | Very Serious | N/A | None | Serious | None | n=20  11.2 (2.9) | n=20  14.4 (3.3) | -3.2 (-5.1 to -1.3) | + VERY LOW |
| Mean Clinical Opiate Withdrawal Scale (COWS) Score at 2 hours post-treatment | | | | | | | | | | |
| 1  Moshiri 2024 | Randomised trial | Very Serious | N/A | None | None | None | n=20  3.2 (1.6) | n=20  11.3 (3.3) | -8.1 (-9.7 to -6.5) | ++ LOW |

*^Risk of Bias: The overall quality rating was downgraded by one increment if the weighted average of methodological concerns across the study was in one domain and by two levels if the weighted average of methodological concerns across study was in two or more domain^*

*^Inconsistency: The overall quality rating was downgraded by one increment if I2 was ≥ 50% and < 75%, and by two increments if I2 was ≥ 75%; Where only one study contributed data to an outcome no assessment for inconsistency could be made^*

*^Indirectness: All studies matched the pre-specified inclusion criteria. No outcome was downgraded due to indirectness Imprecision: The overall quality rating was downgraded by one increment if the upper or lower 95%CI crossed the lower Minimally Important Difference (MID) or the^*

*^upper or lower 95%CI crossed the upper MID. Default MIDs were set at RRs of 0.75 and 1.25 for dichotomous outcomes, and at 0.5 of the control group mean standard deviation either side of the null line for continuous variables.^*

*^Other considerations: As only one study contributed to all outcomes, publication bias could not be assessed^*

Table S5: The change in individual Opioid Withdrawal Symptoms (OWS) and number of individuals retained in buprenorphine (BPN) treatment stratified by the six pharmacological treatment strategies reported in observational studies

| Pharmacological treatment strategies | All  n=97 (100%) | Opioid Withdrawal Symptoms | | Retained in BPN Treatment | |
| --- | --- | --- | --- | --- | --- |
|  |  | Alleviated  n=64 (66.0%) | Not alleviated n=16 (16.5%) | Retained  n=48 (49.5%) | Not retained n=26 (26.8%) |
| a) Additional doses of buprenorphine as monotherapy | 15 | 11 (73.3) | 4 (26.7) | 6 (40.0) | 1 (6.7) |
| b) Additional doses of buprenorphine in combination with symptomatic agents | 43 | 30 (69.8) | 2 (4.7) | 26 (60.5) | 14 (32.6) |
| c) Additional doses of buprenorphine in combination with both symptomatic agents and full opioid agonists | 16 | 13 (81.3) | 2 (12.5) | 10 (62.5) | 4 (25.0) |
| d) Full opioid agonists in combination with symptomatic agents | 4 | 1 (25.0) | 3 (75.0) | 0 (0.0) | 3 (75.0) |
| e) Full opioid agonists alone | 8 | 3 (37.5) | 2 (25.0) | 2 (25.0) | 1 (12.5) |
| f) Symptomatic agents alone | 11 | 6 (54.5) | 3 (27.3) | 4 (36.4) | 3 (27.3) |

Table S6: Preferred Reporting Items for Systematic Reviews and Meta-Analyses (PRISMA) checklist (2)

| **Section/topic** | **#** | **Checklist item** | **Reported on page #** |  |
| --- | --- | --- | --- | --- |
| **TITLE** | | |  |  |
| Title | 1 | Identify the report as a systematic review, meta-analysis, or both. | 1 |  |
| **ABSTRACT** | | |  |  |
| Structured summary | 2 | Provide a structured summary including, as applicable: background; objectives; data sources; study eligibility criteria, participants, and interventions; study appraisal and synthesis methods; results; limitations; conclusions and implications of key findings; systematic review registration number. | 3 |  |
| **INTRODUCTION** | | |  |  |
| Rationale | 3 | Describe the rationale for the review in the context of what is already known. | 5,6 |  |
| Objectives | 4 | Provide an explicit statement of questions being addressed with reference to participants, interventions, comparisons, outcomes, and study design (PICOS). | 6 (OSM Table 1) |  |
| **METHODS** | | |  |  |
| Protocol and registration | 5 | Indicate if a review protocol exists, if and where it can be accessed (e.g., Web address), and, if available, provide registration information including registration number. | 7 |  |
| Eligibility criteria | 6 | Specify study characteristics (e.g., PICOS, length of follow-up) and report characteristics (e.g., years considered, language, publication status) used as criteria for eligibility, giving rationale. | 7,8 |  |
| Information sources | 7 | Describe all information sources (e.g., databases with dates of coverage, contact with study authors to identify additional studies) in the search and date last searched. | 7 |  |
| Search | 8 | Present full electronic search strategy for at least one database, including any limits used, such that it could be repeated. | OSM Figure S1 |  |
| Study selection | 9 | State the process for selecting studies (i.e., screening, eligibility, included in systematic review, and, if applicable, included in the meta-analysis). | 7,8 |  |
| Data collection process | 10 | Describe method of data extraction from reports (e.g., piloted forms, independently, in duplicate) and any processes for obtaining and confirming data from investigators. | 7,8 |  |
| Data items | 11 | List and define all variables for which data were sought (e.g., PICOS, funding sources) and any assumptions and simplifications made. | 7 (OSM Table S2) |  |
| Risk of bias in individual studies | 12 | Describe methods used for assessing risk of bias of individual studies (including specification of whether this was done at the study or outcome level), and how this information is to be used in any data synthesis. | 8 |  |
| Summary measures | 13 | State the principal summary measures (e.g., risk ratio, difference in means). | 8 |  |
| Synthesis of results | 14 | Describe the methods of handling data and combining results of studies, if done, including measures of consistency (e.g., I^2^) for each meta-analysis. | 8 |  |
| Risk of bias across studies | 15 | Specify any assessment of risk of bias that may affect the cumulative evidence (e.g., publication bias, selective reporting within studies). | 8 |  |
| Additional analyses | 16 | Describe methods of additional analyses (e.g., sensitivity or subgroup analyses, meta-regression), if done, indicating which were pre-specified. | N/A |  |
| **RESULTS** | | | |  |
| Study selection | | 17 | Give numbers of studies screened, assessed for eligibility, and included in the review, with reasons for exclusions at each stage, ideally with a flow diagram. | 9 (OSM Figure S2) |
| Study characteristics | | 18 | For each study, present characteristics for which data were extracted (e.g., study size, PICOS, follow-up period) and provide the citations. | Tables 1 & 2 |
| Risk of bias within studies | | 19 | Present data on risk of bias of each study and, if available, any outcome level assessment (see item 12). | 10, 11, (OSM Figure S3, Table S4) |
| Results of individual studies | | 20 | For all outcomes considered (benefits or harms), present, for each study: (a) simple summary data for each intervention group (b) effect estimates and confidence intervals, ideally with a forest plot. | 10, 11 (OSM Figure S4, Table S5) |
| Synthesis of results | | 21 | Present results of each meta-analysis done, including confidence intervals and measures of consistency. | N/A |
| Risk of bias across studies | | 22 | Present results of any assessment of risk of bias across studies (see Item 15). | 10, 11, (OSM Figure S3, Table S4) |
| Additional analysis | | 23 | Give results of additional analyses, if done (e.g., sensitivity or subgroup analyses, meta-regression [see Item 16]). | N/A |
| **DISCUSSION** | | | |  |
| Summary of evidence | | 24 | Summarize the main findings including the strength of evidence for each main outcome; consider their relevance to key groups (e.g., healthcare providers, users, and policy makers). | 13 |
| Limitations | | 25 | Discuss limitations at study and outcome level (e.g., risk of bias), and at review-level (e.g., incomplete retrieval of identified research, reporting bias). | 13 |
| Conclusions | | 26 | Provide a general interpretation of the results in the context of other evidence, and implications for future research. | 14 |
| **FUNDING** | | | |  |
| Funding | | 27 | Describe sources of funding for the systematic review and other support (e.g., supply of data); role of funders for the systematic review. | 2 |

References

1. Wesson DR, Ling W. The Clinical Opiate Withdrawal Scale (COWS). Journal of Psychoactive Drugs. 2003;35(2):253-9.

2. Moher D, Liberati A, Tetzlaff J, Altman DG, The PG. Preferred Reporting Items for Systematic Reviews and Meta-Analyses: The PRISMA Statement. PLOS Medicine. 2009;6(7):e1000097.
